# Supplementary material for: Micro-environmental sensing by bone marrow stroma identifies IL-6 and TGFβ1 as regulators of hematopoietic ageing
Source: Nat Commun. 2020 Aug 14;11:4075. doi: 10.1038/s41467-020-17942-7 (PMC7427787; doi:10.1038/s41467-020-17942-7)
Supplement: Supplementary file 1 — Supplementary Information [file 41467_2020_17942_MOESM1_ESM.pdf]

Micro-environmental sensing by bone marrow stroma identifies IL-6 and TGFβ1 as regulators of hematopoietic ageing.

Valletta et al.

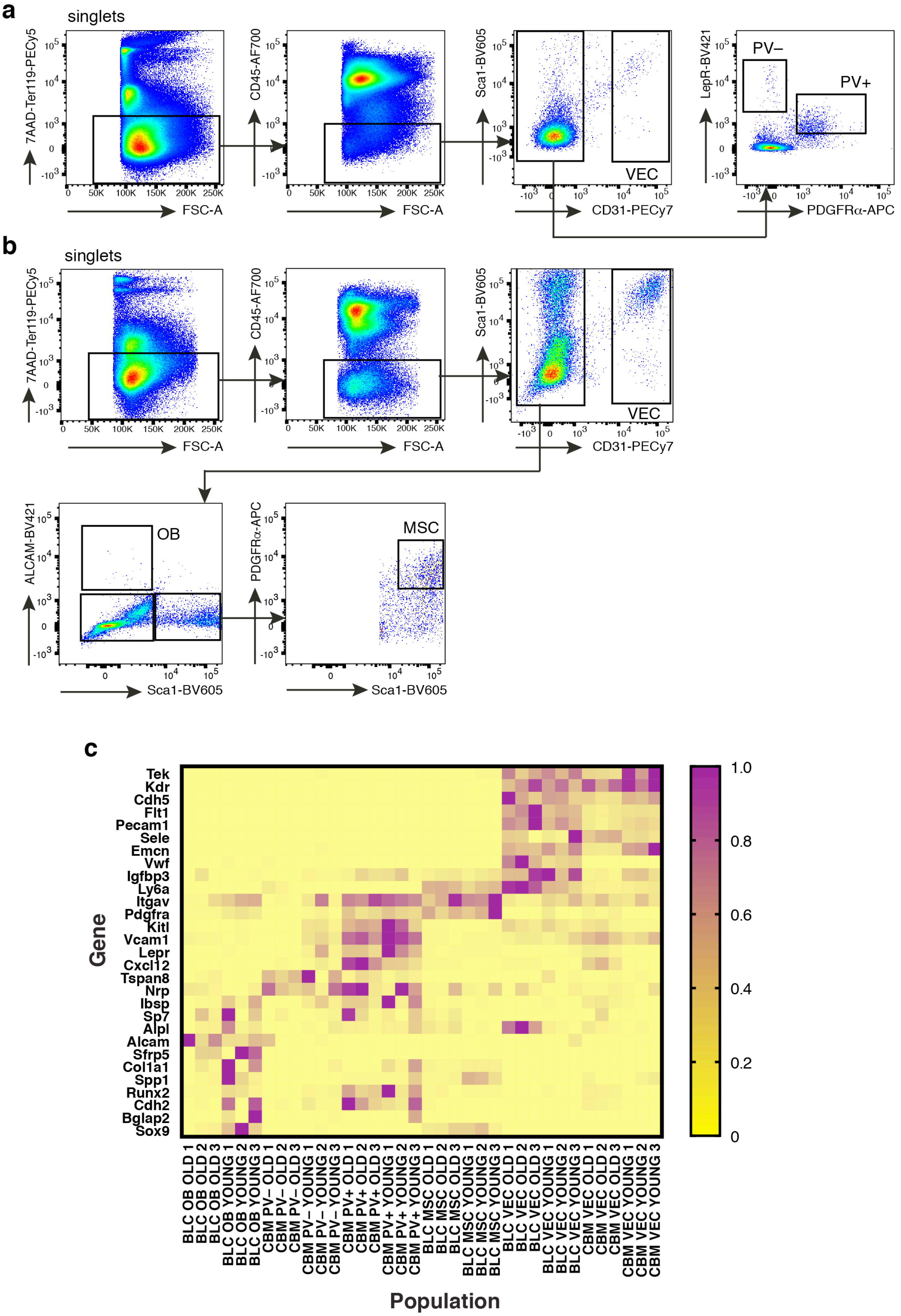

Supplementary Figure 1

Supplementary Figure 1. Isolation and characterization of stromal cell types.

a) Gating strategy for the isolation of central bone marrow (CBM) stromal cells. VEC: vascular endothelial cells; PV–: LepR+PDGFRα– perivascular cells. PV+: LepR+PDGFRα+ perivascular cells.

b) Gating strategy for the isolation of bone lining (BL) stromal cells. VEC: vascular endothelial cells; OB: osteoblasts; MSC: mesenchymal stromal cells.

c) Heatmap of the expression of the indicated marker genes in young and old sorted bone marrow stromal cell populations. Data were normalized and scaled for each gene individually.

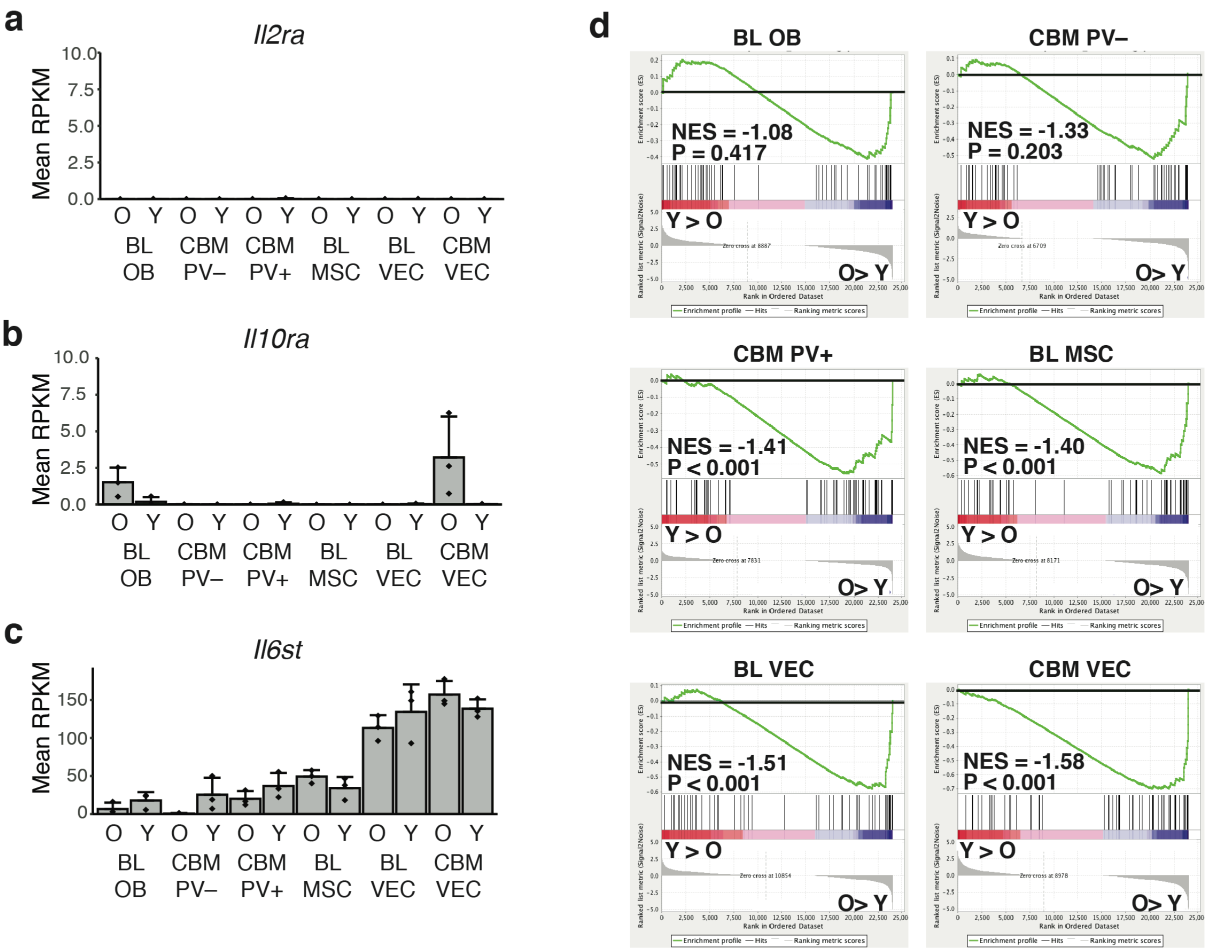

Supplementary Figure 2

Supplementary Figure 2. IL-6 signaling in aged stromal cells.

a-c) Expression of genes encoding the indicated cytokine receptor subunits in young and old stromal cell populations measured by RNA sequencing. The values represent the mean ± s.d. of 3 biological replicates/population.

d) GSEA analysis comparing the expression of IL-6 induced genes (MSigDb gene set M14344) between young and old bone marrow stromal cells for the indicated cell populations. The normalized enrichment score (NES) and P-value are shown.

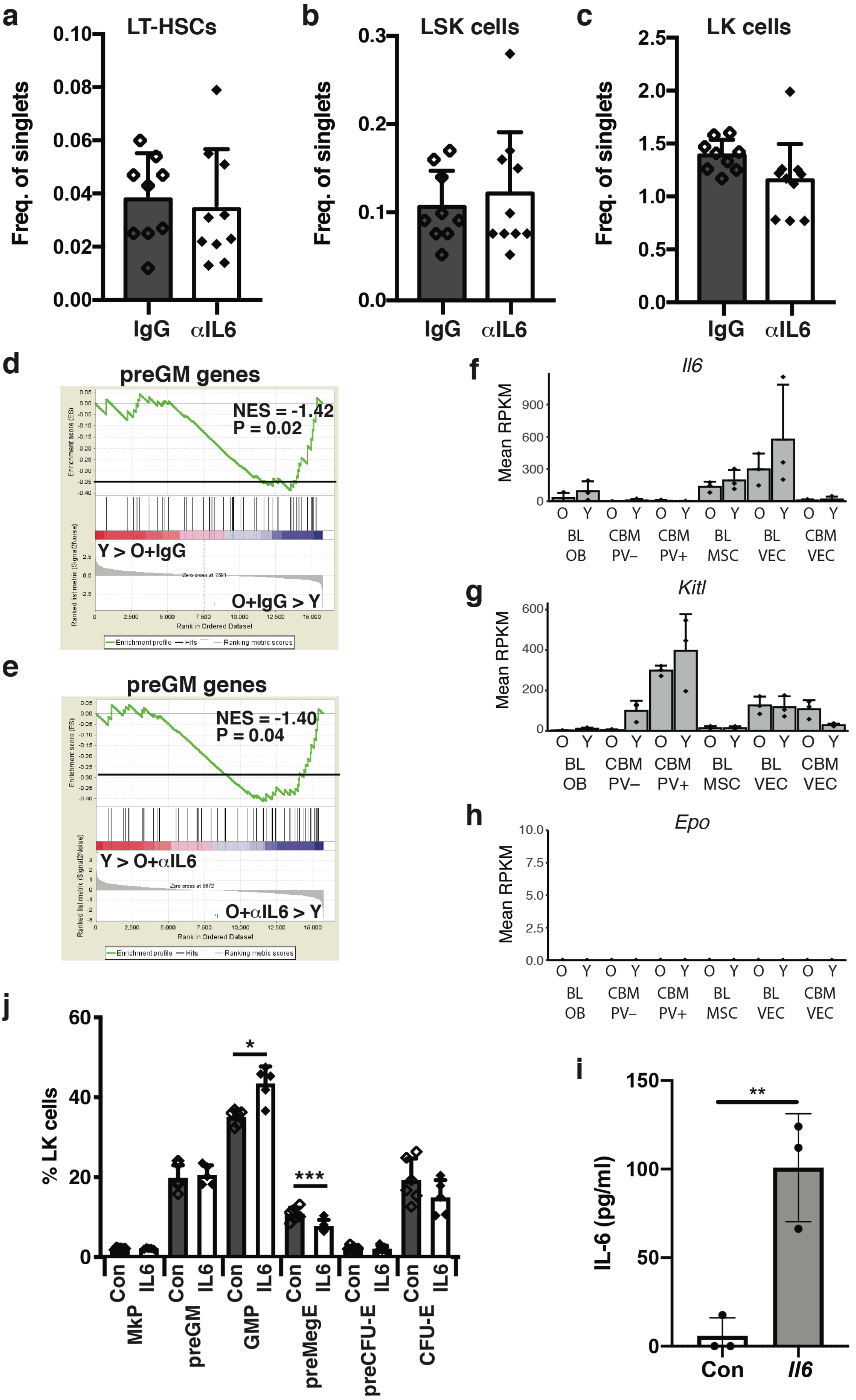

Supplementary Figure 3

Supplementary Figure 3. Effect of IL-6 on HSCs and myeloid progenitors.

a-c) Bar graph showing the number of LT-HSCs (a), LSK (b) and LK (c) cells isolated from BM of old mice after injection of control IgG (N=9) or anti-IL-6 antibody (N=10) as frequency of live single cells. Data are from 5 independent experiments and are shown as mean ± s.d. Differences between conditions were not significant (two-tailed, unpaired Student's t-test).

d) GSEA analysis comparing expression of preGM-specific genes in preCFU-E cells isolated from BM of young mice and old mice after injection of control IgG. The normalized enrichment score (NES) and P-value are shown.

e) GSEA analysis comparing expression of preGM-specific genes in preCFU-E cells isolated from BM of young mice and old mice after injection of anti-IL-6 antibody. The normalized enrichment score (NES) and P-value are shown.

f) Expression of the gene encoding IL-6 in young and old stromal cell populations measured by RNA sequencing. The values represent the mean ± s.d. of 3 biological replicates/population.

g) Expression of the gene encoding KitL/SCF in young and old stromal cell populations measured by RNA sequencing. The values represent the mean ± s.d. of 3 biological replicates/population.

h) Expression of the gene encoding Epo in young and old stromal cell populations measured by RNA sequencing. The values represent the mean ± s.d. of 3 biological replicates/population.

i) Quantification by ELISA of IL-6 protein in young mice injected with empty pCMV-entry vector (Control, N=3) or pCMV6-Entry vector containing IL-6 cDNA (IL-6, N=3). Data are from 2 independent experiments. Values show mean ± s.e.m. . ** P<0.01 (two-tailed unpaired Student's t-test). Exact P value: 0.0069.

j) Quantification of myelo-erythroid progenitors from young mice hydrodynamically injected with empty pCMV-entry vector (Control, N=6) or pCMV6-Entry vector expressing *Il6* cDNA (*Il6*, N=5). Data are from 2 independent experiments. Values are mean ± s.d. * P<0.05; ***P<0.001 (two-tailed unpaired Student's t-test). Exact P values: MkP: 0.50; preGM: 0.67; GMP: 0.002; preMegE: 0.01; preCFU-E: 0.72; CFU-E: 0.19.

Source data are provided as a source data file.

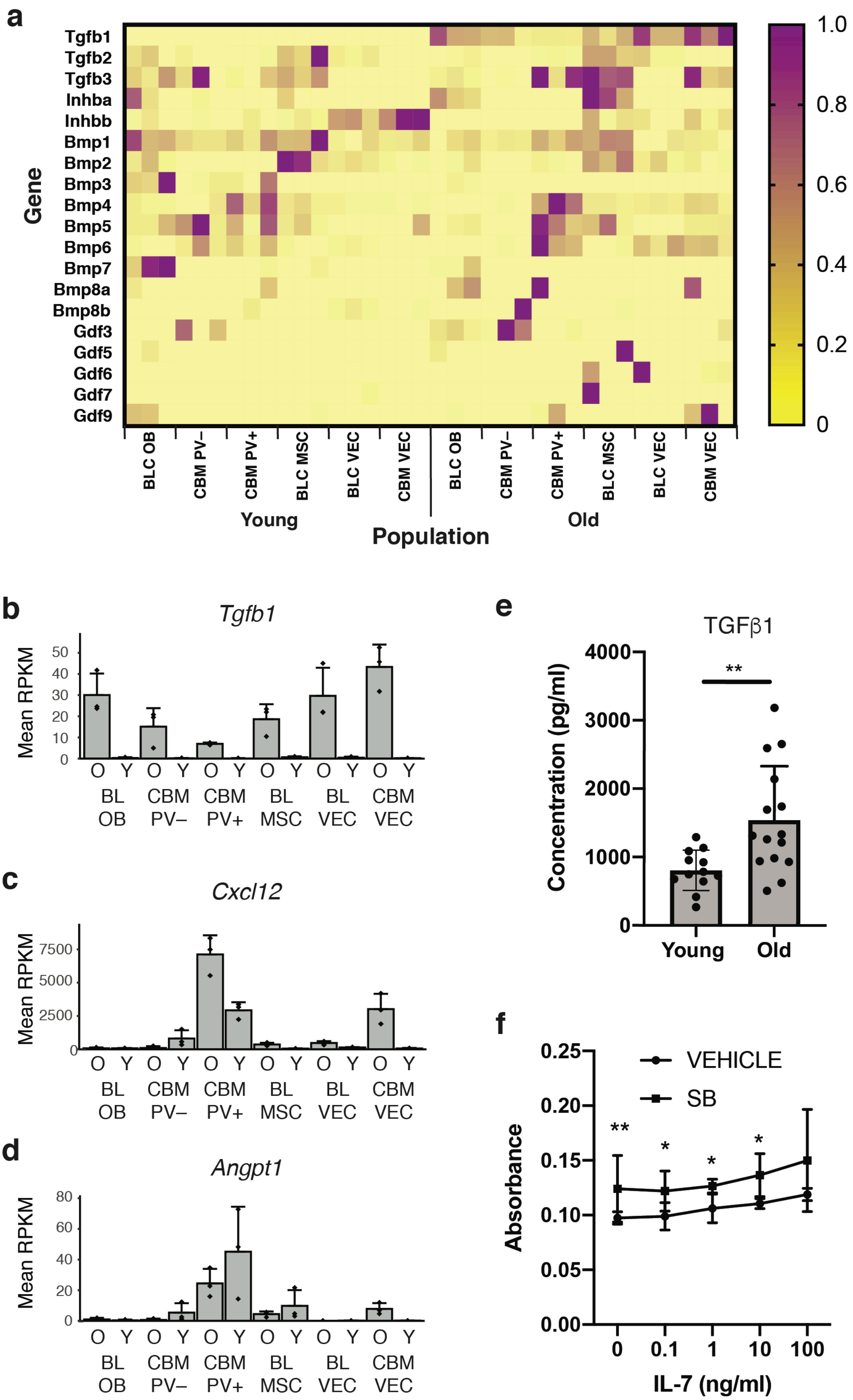

Supplementary Figure 4

Supplementary Figure 4. TGFβ signaling in aged bone marrow.

c) Heatmap of the expression of genes encoding the indicated TGFβ/activin/GDF family ligand genes in young and old sorted bone marrow stromal cell populations. Data were normalized and scaled for each gene individually.

b-d) Expression of the genes encoding TGFβ1 (b), Cxcl12 (c) and Angiopoietin-1 (d) in young and old stromal cell populations measured by RNA sequencing. The values represent the mean ± s.d. of 3 biological replicates/population.

e) Quantification by ELISA of TGFβ1 protein in bone marrow supernatant obtained from young (N=12) and old mice (N=15). Data are from 2 independent experiments. Values show mean ± s.e.m. ** P<0.01 (two-tailed unpaired Student's t-test). Exact P value: 0.005.

f) Proliferation of early B precursor cells determined by MTS assay. The absorbance at 490nm was recorded after 48 hours. Cells were isolated from vehicle (VEHICLE, N=5) and SB-treated (SB, N=7) old mice (3 independent experiments) cultured with different concentrations of mIL-7 (0.1, 1, 10, and 100ng/ml, as indicated). Values show mean ± s.e.m. * P<0.05; ** P<0.01 (two-tailed unpaired Student's t-test, no correction for multiple testing). Exact P values: 0ng/ml: 0.0051; 0.1ng/ml: 0.0341; 1ng/ml: 0.0101; 10ng/ml: 0.0480; 100ng/ml: 0.3290.

Source data are provided as a source data file.
