# Supplementary material for: Micro-environmental sensing by bone marrow stroma identifies IL-6 and TGFβ1 as regulators of hematopoietic ageing
Source: Nat Commun. 2020 Aug 14;11:4075. doi: 10.1038/s41467-020-17942-7 (PMC7427787; doi:10.1038/s41467-020-17942-7)
Supplement: Supplementary file 3 — Reporting Summary [file 41467_2020_17942_MOESM3_ESM.pdf]

nature research

Corresponding author(s): *Double-blind peer review submissions: write DBPR and your manuscript number here instead of author names.*

Last updated by author(s): *YYYY-MM-DD*

Reporting Summary

Nature Research wishes to improve the reproducibility of the work that we publish. This form provides structure for consistency and transparency in reporting. For further information on Nature Research policies, see Authors & Referees and the Editorial Policy Checklist.

Statistics

For all statistical analyses, confirm that the following items are present in the figure legend, table legend, main text, or Methods section.

n/a	Confirmed	
☐	☒	The exact sample size (*n*) for each experimental group/condition, given as a discrete number and unit of measurement
☐	☒	A statement on whether measurements were taken from distinct samples or whether the same sample was measured repeatedly
☐	☒	The statistical test(s) used AND whether they are one- or two-sided<br>*Only common tests should be described solely by name; describe more complex techniques in the Methods section.*
☒	☐	A description of all covariates tested
☒	☐	A description of any assumptions or corrections, such as tests of normality and adjustment for multiple comparisons
☒	☐	A full description of the statistical parameters including central tendency (e.g. means) or other basic estimates (e.g. regression coefficient) AND variation (e.g. standard deviation) or associated estimates of uncertainty (e.g. confidence intervals)
☒	☐	For null hypothesis testing, the test statistic (e.g. *F*, *t*, *r*) with confidence intervals, effect sizes, degrees of freedom and *P* value noted<br>*Give P values as exact values whenever suitable.*
☒	☐	For Bayesian analysis, information on the choice of priors and Markov chain Monte Carlo settings
☒	☐	For hierarchical and complex designs, identification of the appropriate level for tests and full reporting of outcomes
☒	☐	Estimates of effect sizes (e.g. Cohen's *d*, Pearson's *r*), indicating how they were calculated

Our web collection on statistics for biologists contains articles on many of the points above.

Software and code

Policy information about availability of computer code

Data collection	Flow Cytometry data: BD FACSDiva 8.0.1.
Data analysis	Flow Citometry data analysis: FlowJo 10.3<br>Data visualzation and statistical comparisons with t-test: GraphPad Prism 7.0.

For manuscripts utilizing custom algorithms or software that are central to the research but not yet described in published literature, software must be made available to editors/reviewers. We strongly encourage code deposition in a community repository (e.g. GitHub). See the Nature Research guidelines for submitting code & software for further information.

Data

Policy information about availability of data

All manuscripts must include a data availability statement. This statement should provide the following information, where applicable:

- Accession codes, unique identifiers, or web links for publicly available datasets
- A list of figures that have associated raw data
- A description of any restrictions on data availability

All unique materials used are available from the authors upon reasonable request.

Field-specific reporting

Please select the one below that is the best fit for your research. If you are not sure, read the appropriate sections before making your selection.

☒ Life sciences ☐ Behavioural & social sciences ☐ Ecological, evolutionary & environmental sciences

Life sciences study design

All studies must disclose on these points even when the disclosure is negative.

Sample size	No statistical methods were used to predetermine the experimental sample size.
Data exclusions	Not applicable
Replication	For all figures, multiple independent experiments were performed
Randomization	Young mice were 2-3 months old and old mice were 24-25 moths old. Only female mice were used for experiments. When multiple experimental groups were analysed, mice were allocated so that each group was evenly matched for age range. No statistical methods were used to predetermine the experimental sample size.
Blinding	Blinding was not performed.

Reporting for specific materials, systems and methods

We require information from authors about some types of materials, experimental systems and methods used in many studies. Here, indicate whether each material, system or method listed is relevant to your study. If you are not sure if a list item applies to your research, read the appropriate section before selecting a response.

Materials & experimental systems

n/a	Involved in the study	
☐	☒	Antibodies
☐	☒	Eukaryotic cell lines
☒	☐	Palaeontology
☐	☒	Animals and other organisms
☒	☐	Human research participants
☒	☐	Clinical data

Methods

n/a	Involved in the study	
☒	☐	ChIP-seq
☐	☒	Flow cytometry
☒	☐	MRI-based neuroimaging

Antibodies

Antibodies used	All antibodies used in the study were obtained from commercial vendors. Their details (Supplier, conjugate, catalogue number and application) are described in Supplementary Table 1.
Validation	All antibodies were validated by their manufacturers for the application (flow cytometry) and species (mouse) used in this study. In addition, all antibodies used were individually titrated before their use to identify their optimal working concentration. In all the experiments fluorescence-minus-one (FMO) were included and when possible, staining panels included internal controls (validated negative and positive populations) in order to validate antibody specificity.

Eukaryotic cell lines

Policy information about cell lines

Cell line source(s)	OP9 stromal cells for supporting B cells differentiation in vitro were provided by...
Authentication	OP9 cell line has a distinct morphology and can uniquely promote B cell differentiation from hematopoietic/progenitor cells. All of these properties have been validated.
Mycoplasma contamination	OP9 cells were tested for mycoplasma contamination by PCR and found negative in all tests.
Commonly misidentified lines (See ICLAC register)	No commonly misidentified lines were used.

Animals and other organisms

Policy information about studies involving animals; ARRIVE guidelines recommended for reporting animal research

Laboratory animals	Young female mice (2-3 months old) and old mice (24-25 moths old) were used for experiments.<br>All mouse strains used are reported in Methods: "Animals"<br>Wild type B6SJLCD45 (CD45.1)

	Wild type C57BL/6 (CD45.2 or CD45.1)<br>Vwf-tdTomato/Gata1-eGFP double reporter mice (CD45.2)
Wild animals	Not applicable
Field-collected samples	Not applicable
Ethics oversight	All experimental procedures and mouse breeding and maintenance were in accordance with UK Home Office regulations. All experiments were approved by the Oxford University Clinical Medicine Ethical Review Committee.

Note that full information on the approval of the study protocol must also be provided in the manuscript.

Flow Cytometry

Plots

Confirm that:

☒ The axis labels state the marker and fluorochrome used (e.g. CD4-FITC).

☒ The axis scales are clearly visible. Include numbers along axes only for bottom left plot of group (a 'group' is an analysis of identical markers).

☒ All plots are contour plots with outliers or pseudocolor plots.

☒ A numerical value for number of cells or percentage (with statistics) is provided.

Methodology

Sample preparation	Bone marrow and peripheral blood from wild type and transgenic mice, as well as in vitro cultures generated by mouse bone marrow cells, were prepared into single cell suspension in PBS medium supplemented with 5% fetal calf serum. All samples were incubated with Fc-block/CD16/32 PE-Cy7 prior to staining with monoclonal antibodies. For details, see Methods.
Instrument	For data collection the following instruments were used:<br>BD FACAriaII, BD LSRII and BD LSR Fortessa X-20.
Software	For data collection for all experiments, BD FACSDiva was used. For Data analysis FlowJo 10.3 was used.
Cell population abundance	LT-HSCs: 7-25% of total BM<br>Hematopoietic progenitors: 7-15% of BM LK population<br>BL stromal cells: 10-15% of BM viable Ter119- population<br>CBM stromal cells: 3-5% of BM viable Ter119- population
Gating strategy	FSC-A/SSC-A was used for gating mononuclear cells. FSC-A/FSC-H was used for gating on singlets. 7AAD-negative cells were gated out to exclude non-viable cells. Where relevant , lineage-cocktail was used for gating out Lineage-positive cells. In peripheral blood analysis, mature cells were gated considering positive and negative markers. When possible, phenotypic definition of each population included both positive and negative markers. Specific gating strategies are included in Methods and exemplified in Figures.

☒ Tick this box to confirm that a figure exemplifying the gating strategy is provided in the Supplementary Information.
