# Supplementary material for: Micro-environmental sensing by bone marrow stroma identifies IL-6 and TGFβ1 as regulators of hematopoietic ageing
Source: Nat Commun. 2020 Aug 14;11:4075. doi: 10.1038/s41467-020-17942-7 (PMC7427787; doi:10.1038/s41467-020-17942-7)
Supplement: Supplementary file 4 — Description of Additional Supplementary Files [file 41467_2020_17942_MOESM4_ESM.pdf]

Description of Additional Supplementary Files

Supplementary Data 1.
The genes differentially expressed at an adjusted P-value < 0.1 between young and old cells from the indicated bone marrow stromal cell populations measured by RNA sequencing. For each population 3 biological replicates were generated.

Supplementary Data 2.
Enrichment of Metacore process networks during ageing in the indicated bone marrow stromal cell populations measured by RNA sequencing. For each population 3 biological replicates were generated.

Supplementary Data 3.
Details of mouse antibodies and viability dyes used for each flow cytometry staining panel.

Supplementary Data 4.
MPP2, MPP4, preCFU-E and preGM specific gene sets used for Gene Set Enrichment Analysis.
